# Icteroid Fever

**Published:** 1885-12-20

**Authors:** B. F. Humphreys

**Affiliations:** Hawkins, Texas


### Southern Medical Record.

EDITORS:
THOMAS S. POWELL, M.D. R. C. WORD, M.D.

*R. C. WORD, M.D., Managing Editor.*

☞ All Communications and Letters on Business connected with the RECORD must be addressed to the Managing Editor.

VOL. XV. ATLANTA, GA., DECEMBER 20, 1885. No. 12.

### ORIGINAL AND SELECTED ARTICLES.

### ICTEROID FEVER.

A BRIEF STUDY OF ITS PATHOLOGY AND TREATMENT.

BY B. F. HUMPHREYS, M. D., HAWKINS, TEX.

There are, perhaps, many readers of the SOUTHERN MEDICAL RECORD who have encountered this malady under the name of *malarial hæmaturia*, *hemorrhagic malarial fever*, *black jaundice*, *swamp fever*, *the yellow disease*, etc. The latter name is more commonly applied to it by the people than any of the other titles. In fact, it expresses one of the leading symptoms more clearly than any of the other names. It is emphatically *the yellow disease*, for which I have appropriated the classical cognomen of ICTEROID, from the Greek *ixtegos*, yellow, and *eidos*, like, resembling. Icteroid fever is, therefore, a fever which resembles yellow fever in many respects not necessary to enumerate here.

I have elsewhere* expressed my views in regard to the pathology and treatment of this fever, which is becoming more prevalent in this State every year, while it is, perhaps, equally prevalent and fatal in other Southern States. There is no disease in this State which more loudly calls for our earnest study and careful investigation.

* Nashville Journal of Medicine and Surgery, March, 1874; May, 1876; October, 1885; Medical Brief, February, 1885.

As I have recently had an attack of this dreadful malady in my own person, and as I have resided where it has prevailed for the last two decades, and have often encountered it, I desire to write something practical and acceptable to the readers of the RECORD.

It is, without doubt, a disease of the hemorrhagic diathesis ; and, in a former contribution on the subject* I used the term hemorrhagic ; but, as the disease cannot exist without the hemorrhage, it is not necessary to continue this term. The hæmaturia, which consists of a dark excretion from the kidneys, resembling beef-brine, is really one of the most conclusive pathognomic symptoms of this fever. Without this hæmaturia, the disease under consideration cannot exist, whatever other symptoms may be necessary to establish the diagnosis. This so-called hæmaturia or secretion from the kidneys does not coagulate, and a blister drawn upon any part of the body fills with this dark blood instead of serum. A young physician, whom I met a few days ago, expressed his surprise at this fact, which he had noticed in a case that he had recently treated.

This is the stepping-stone to the true pathology of this terrible malady. Why does a blister fill with the dark blood, so-called, instead of serum ? Evidently, because the blood is changed. The malarial poison—whatever that may be—has entirely changed the healthy condition of the blood, perhaps by destroying the normal *cohesion* existing between the corpuscular elements, wherefore the red blood corpuscles, no longer having any affinity for the other corpuscular elements, are eliminated through the kidneys as a waste product. This is not mere presumption, for whoever has carefully observed this disease, and its destructive effects, must be convinced that the excretion from the kidneys is something more, or something else, than a mere " bilurea "—bilirubine and biliverdine—as claimed by some writers ; and Chemistry establishes the fact that the red blood corpuscles, phosphates, and some other important elements of the blood are thus eliminated.

The malarial poison having produced such remarkable changes in the blood, it should not be surprising that the disease is so often fatal in spite of the most careful and scientific treatment ; and the physician who is searching for a specific or infallible remedy for this fever has never carefully investigated the first principles of its pathology. Upon the fatal termination of several cases of this fever, recently, a young physician remarked to me that he must have a better and more successful treatment. He is of a practical turn

* **Nashville Journal of Medicine and Surgery, March, 1874.**

of mind, and wants to realize success in the face of insurmountable difficulties; but, if we shall only think for a moment, we shall see how utterly impossible it is to cure all these cases.

A young lady, who was recently stricken down with this frightful malady, and finally succumbed to its effects, had a light attack of malarial fever some two or three weeks preceding this last attack. In my own attack, I had no fever of any kind during the malarial season; had been in perfect health until stricken down with hæmaturia—something remarkable in the history of this fever, as it has heretofore been considered impossible for this disease to exist or occur in any person who has not had repeated attacks of malarial fever previous to the advent of this malady.

On the 18th of October, the young lady referred to had a light fever, but was up all day attending to household duties. At night she was given an active and powerful antibilious course, wherefore she complained much of its griping, painful effects—said it was killing her! A dose of opium was given her, and she slept quietly until morning, when she arose and proceeded to her household duties, but returned to her bed. A capsule, containing about five grains of quinine, was now given to her, as her temperature was below 100° Fah. Soon after taking the quinine she had a chill, which was followed by a desire to micturate, when it appeared the pathognomonic excretion from the kidneys was present. Her stomach was fearfully involved from the time she complained of the griping of the purgative medicine; constant nausea and vomiting; in consequence of which she refused everything in the way of nourishment. Her temperature soon reached 105° Fah., and continued so for two days. This young lady had had this fever several times before, some years ago, and her stomach was always involved to an alarming degree. The remedies which previously relieved the nausea and retching were again resorted to, and relieved this trouble in a great degree. A few spoonfuls of crushed ice were given to her from time to time, when complaining of nausea, and always gave relief for the time. This seemed to be very acceptable to the patient.

The constant nausea prevented the patient from taking sufficient nourishment to renew the deteriorated blood, the hæmaturia continuing for three days; the skin was the most deeply colored of any case I had ever seen, and looked as if it had been painted with yellow chrome. She was rational until the last evening of her illness, which lasted only six days, during which she rarely ever spoke above a whisper. Notwithstanding the urinary secretions had cleared up to the color of the tincture of the chloride of iron hopes were entertained of her final recovery, but she suddenly fell into a fatal collapse, refusing stimulants and hypodermic treatment, wherefore she sank into the arms of Death, on the evening of the sixth day of her illness, through exhaustion.

Reviewing the above results, the question naturally arises: Why did the treatment which had as often relieved others fail in this case? The answer can readily be given. In the first place, the attack was unusually violent; the skin was more deeply colored; the hæmaturia was more exhausting; the stomach was more seriously involved; the red blood corpuscles were *all*, perhaps, eliminated, together with other important elements of the blood, the patient could not reasonably survive longer. Those cases which recover are less violent, not so complicated, take the requisite amount of nourishment to renew the progressive waste, and likewise take stimulants to tide them over immediate danger. Hence, the reasonable conclusion why some recover and others do not under the same treatment, is simply this: Some cases, as the one described above, are so violent from the onset, the blood being so completely changed, or broken down, the patient refusing the necessary nourishment to restore the blood, it is evident that recovery is impossible, wherefore it is not necessary nor advisable to discard a treatment which has proven successful in a large majority of cases, because, forsooth, some terminate fatally.

It may not be amiss, however, to study the pathology of this disease more carefully, before seeking remedies for the irreparable. A man in this village was stabbed in the region of the heart, a few weeks ago, and he had bled almost to death before the artery was ligated. He survived, without nourishment or transfusion, until the next evening, and died of exhaustion. This result was no more surprising to the physician acqainted with cause and effect, than the death of the young lady referred to above; and, *vice versa*, the death of the latter should be regarded as unavoidable as the former.

A sound pane of glass may bear a somewhat heavy pressure without breaking. A piece of glass fractured nearly in two will bear a very light pressure without being broken. I have taken this simple fact to illustrate the manner in which a serious result may follow a lack of knowledge, or caution, on the part of the physician or others who recklessly administer powerful drugs. If, by looking at a patient's tongue, skin, countenance, feeling the pulse, etc., we could tell the real condition of the blood, as readily as we can recognize the frail condition of the fractured glass, we would, many times, act more cautiously, and not destroy the feeble cohesion existing in the elements of the blood, which may be severed by a few heavy doses of some powerful drug, when the patient is already in a condition for some drastic or powerful medication to precipitate an attack of this much-dreaded malady, which otherwise might be avoided.

It has been claimed by some writers, who seem to be only partially acquainted with the pathology of this fever, that quinine produces the terrible results following an attack of this disease; but, if the pathological views expressed in the preceding part of this paper are correct, we may readily see that such a conclusion is not strictly correct. As only a light pressure may sever the fractured glass, just so a few doses of drastic purgatives, or heroic dose of quinine, or other powerful drugs, may precipitate an attack of malarial hæmaturia, by destroying the feeble cohesion existing between the corpuscular elements of the blood, when the patient is already upon the verge of this disease, which, if known, might have been avoided by using mild remedies, and thus save the patient from the terrible ordeal of passing through an attack of this malady, which so often ends in death.

It is, therefore, very important that we should handle this "harp of a thousand strings" with extreme caution. Except in certain well known maladies, which require very active treatment by large doses of powerful drugs, it is nearly always best to use mild remedies, which can be frequently repeated; and, to accomplish this end, I know of nothing better and more satisfactory to the physician and to the patient than a class of remedies known as *Parvules*, manufactured by the well known firm of W. R. Warner & Co., of Philadelphia. Two parvules of calomel, containing one-twentieth of a grain, each, given every hour until ten parvules are administered, usually produces a decided impression upon the liver, followed by as copious bilious discharges as if ten grains of calomel had been given, while only one-half grain of the drug has been given. They may be taken by children with the same impunity as by adults, though a less number will suffice for children. There is no physician, perhaps, who has witnessed an attack of icteroid fever, after administering a drastic purgative course, or quinine heroically, or other powerful drugs, who would not recall the treatment, if he could, and use milder remedies, such as I have already suggested.

If the pathological views already advanced in this article are correct, it must be apparent now that the treatment advised by many practitioners is very irrational. If the blood-changes alluded to have already taken place, there can be no reason for giving ergot and similar agents to prevent the elimination of this wasteproduct. It would be far more rational to give those remedies which favor the elimination of this waste-product, and experience proves this to be the most successful treatment. Many physicians advise ergot, tannin, gallic acid, and similar drugs, with a view to check the so-called hemorrhage, which really should be encouraged, or expelled from the circulation. A teaspoonful of the sweet spirits of nitre, well diluted with water, followed in an hour with a tablespoonful of a saturated solution of the chlorate of potassium, and an hour after this a swallow of Cognac brandy, in which should be suspended a teaspoonful of willow charcoal, the mixture diluted and sweetened to suit the taste ; these remedies should be continued in this way until the hæmaturia ceases, and all the symptoms improve, when the interval between the remedies may be lengthened, and the drugs given as often as may seem necessary.

As the patient, sometimes, takes a dislike to some certain remedy, it may be well to have some other to take its place, and I know of nothing better than the tincture of the chloride of iron, in ten to twenty-drop dose, every two, three or four hours, as may seem necessary, until the hæmaturia ceases and the urine clears up, when it may be given three or four times a day until the patient makes a complete recovery. In fact, it may be given in connection with the other remedies from the onset, and it will always be found a useful agent in the treatment of this fever.

For the extreme nausea and vomiting, a few spoonfuls of crushed ice often allays the intolerable nausea for the time being—which has to be repeated as often as this trouble occurs. Should the ice fail—and it rarely does—five grains of the oxalate of cerium, one-fourth grain of morphine, with three to five drops of pure creasote, placed in a No. 1 capsule and filled with the subnitrate of bismuth, should be given every three or four hours until the nausea and retching is controlled.

Let us beware of curious polypharmacy and much drugging in this, as well as in all other maladies, always watching for the first revolt of the stomach, which is a reliable sentinel. As soon as the stomach will retain nourishment, it should be administered freely in a liquid form—such as beef-tea properly made, animal and vegetable soups, sweet milk, or milk and brandy mixed.

The physician, or an experienced, intelligent nurse, should be by the bedside day and night, watching for the first symptoms of collapse, which should be combatted with brandy—the imported Cognac is to be preferred—or milk and brandy mixed. Two or three drops of nitro-glycerine may be placed upon the tongue in a great emergency, when the collapse is coming on rapidly, or atropia may be used hypodermically when the patient fails to swallow, and the brandy may be thus used also ; or the stimulants and nourishment may be administered by enemas when the stomach rejects these remedies. While combatting collapse with internal stimulants, the surface should be briskly rubbed with dry mustard, or with a powerfully stimulating liniment, which may be applied along the spine and to the extremities. Electricity, if convenient, may also be brought into requisition, in order to aid in tiding the patientover the collapse.

In conclusion, let us beware of blisters and quinine in this malady, as both are apparently injurious. The blister adds only to the sufferings of the patient, while the quinine seems to increase the rigors and aggravate all the symptoms. It was supposed to be *the* remedy in this disease a decade ago ; but, as the malady now manifests itself, experience has proven that quinine is inadmissible, and that very few patients recover under its use.

Every effort should be made to encourage the patient to take liquid nourishment to renew the blood. Failing to get the patient to take the nourishment per orem, the only hope for life is to administer the remedies or alimentation by enemas. Mustard plasters may be used for a few minutes at a time—say five to ten minutes—over the stomach and kidneys, instead of blisters. Tonics and stimulants should be given upon the decline of the disease, and continued until the health is fully restored.

A well-to-do farmer, who resided a few miles North of this place, had a severe attack of hæmaturia about seventeen years ago, his urinary organs being damaged thereby. He had a renewed attack a few weeks ago, which proved fatal. I do not know any of the details of his case. The safest and surest propylaxis, therefore, is a removal from the malarial to a healthy climate.

The thoughts presented in this paper are the result of a sad experience—the loss in this dark malarial abode of nearly all who were nearest and dearest to me by the ties of Nature, and almost the sacrifice of my own life. I hope this costly experience may be of some value to my co-laborers.

---

**An Aerial Railway and Car** has been patented by Mr. Andrew J. Morrison, of Buffalo, N. Y. It consists of a wire cable supported at intervals by balloons anchored to the earth, a car being suspended from the cable and made to travel thereon by its own gravity, the balloons being arranged to raise and lower the cables so as to give them the proper inclination.—*Scien. American.*

